# Training as a psychiatrist during a pandemic

**DOI:** 10.1192/j.eurpsy.2021.213

**Published:** 2021-08-13

**Authors:** C. Migchels

**Affiliations:** University Psychiatric Centre, KULeuven, Kortenberg, Belgium

## Abstract

Training is a crucial time in any psychiatrist’s career. It’s a time when you are faced with new responsibilities and challenges, both professionally and personally, and senior and peer support are indispensable. The Covid-19 pandemic has had an impact on all the essential needs of trainees: supervision, theoretical training, clinical care and contact with both patients and colleagues have been put under pressure and are no longer the self-evident part of training that they were before. Trainees are facing an unprecedented balancing act between their duties as physicians, their training needs and their dedication to the often vulnerable patients under their care, all whilst dealing with the personal consequences of the pandemic and the restrictions that have been put in place. Based on the experiences of the European Federation of Psychiatric Trainees (EFPT) we will have a look into what has changed in the lives of trainee psychiatrists globally since the start of the pandemic, the difficulties that have occured and the lessons that have been learned. Examples of ways of dealing with the above mentioned challenges and opportunities for the future will be discussed.

**Disclosure:**

No significant relationships.

